# A Quasi-Michaelis–Menten Mechanism Explains the Nonlinear Relationship between Transition Metals and PM_2.5_ Oxidative Potential Measured by the Dithiothreitol (DTT) Assay

**DOI:** 10.1021/acs.est.6c09293

**Published:** 2026-09-19

**Authors:** Hanzhe Chen, Yuhuang Cheng, Kezheng Liao, Manfei Lin, Tao Li, Jian Zhen Yu

**Affiliations:** † Division of Environment and Sustainability, 12589 The Hong Kong University of Science and Technology, Hong Kong 999077, China; ‡ Department of Chemistry, 567841 The Hong Kong University of Science and Technology, Hong Kong 999077, China; § Institution of Atmospheric Environment, 563606 Jiangsu Provincial Academy of Environmental Science, Nanjing, Jiangsu 210036, China; ∥ School of Environmental Science and Engineering, Shandong University, Qingdao, Shandong 266237, China

**Keywords:** particulate matter, oxidative potential, reactive oxygen species, transition metals, Michaelis−Menten kinetics, health impact

## Abstract

Oxidative potential (OP), defined as the capacity of particulate matter (PM) to consume antioxidants and/or generate reactive oxygen species, is increasingly used as a metric for assessing PM toxicity. However, the concentration–response relationship between PM components and OP measured by the dithiothreitol (DTT) assay (OP_DTT_) is often nonlinear, and the underlying mechanism remains poorly understood. Here, we measured OP_DTT_ of laboratory-prepared solutions and ambient PM_2.5_ samples and propose a quasi-Michaelis–Menten mechanism involving DTT, transition metals, and oxygen. The derived equations for copper (Cu) and manganese (Mn) quantitatively reproduced the nonlinear OP_DTT_ response observed in both single-metal standard solutions and ambient samples, as well as the antagonistic effects of Cu–Mn mixtures. For 62 ambient PM_2.5_ samples collected in Beijing over one year, a model based on water-soluble Cu, Mn, and water-soluble organic carbon (WSOC), assuming a linear WSOC contribution, accounted for 98.6% of the measured OP_DTT_. These results support the proposed quasi-Michaelis–Menten mechanism as an explanation for the nonlinear concentration–response relationship and provide a basis for predicting OP_DTT_ from PM_2.5_ chemical composition.

## Graphical abstract

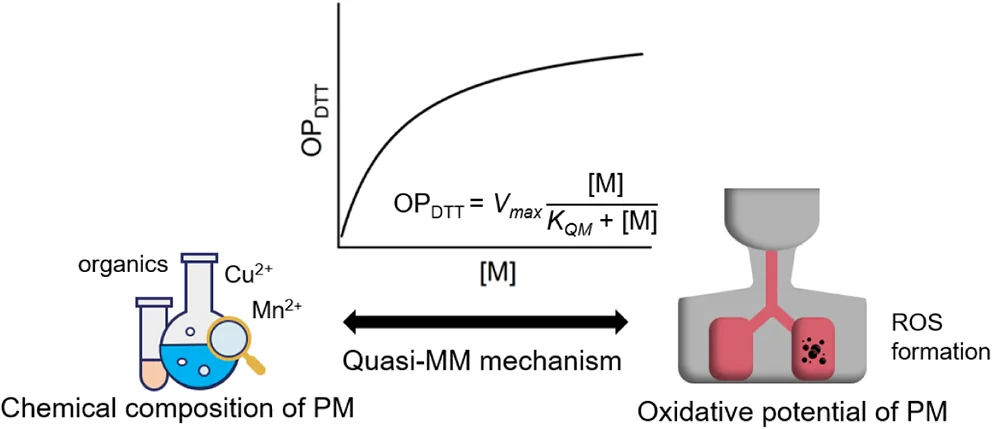

## 1 Introduction

Associations between ambient particulate matter (PM) and adverse health effects (e.g., morbidity and mortality) have been extensively documented over the past decades.^1–4^ Increasing evidence suggests that transition metals (TMs) and redox-active organics in inhaled fine particulate matter (PM_2.5_) can catalyze the formation of reactive oxygen species (ROS) in human lung fluid and thereby induce oxidative stress, resulting in adverse health impacts such as asthma, lung cancer, and cardiovascular diseases.^5–9^ Oxidative potential (OP), defined as the capacity of PM to consume antioxidants or to generate ROS, has therefore emerged as a promising metric of PM toxicity.^6,10^

Dithiothreitol (DTT), a chemical proxy for cellular reductants (e.g., NADH and NADPH),^11^ is one of the widely used acellular assay reagents to determine OP of PM exposure by measuring the depletion rate of DTT (OP_DTT_). Compared with cellular assays, the DTT assay is faster, less expensive, and easier to perform. It is also sensitive to both transition metals and organic compounds, whereas some other acellular assays (e.g., ascorbic assay and electron spin resonance) are often specifically responsive to metals.^6,12^ Epidemiological studies have shown that OP_DTT_ is one of the most health-relevant acellular OP assays, exhibiting strong associations with multiple health endpoints (e.g., acute cardiac and respiratory outcomes).^8,10,13–16^ As a result, a growing number of studies have evaluated the toxicity of PM by measuring OP_DTT_.^17^ To support health-oriented pollution control, many studies have sought to identify the main chemical contributors and sources of OP_DTT_. In most cases, species that correlate strongly with OP_DTT_ are considered as key contributors,^18,19^ and source-specific intrinsic OP_DTT_ values are estimated using multiple linear regression between OP_DTT_ and receptor model-derived sources^8,19–21^ or by direct source apportionment using positive matrix factorization (PMF) modeling.^17,18^ These approaches implicitly assume linear relationships. The assumption conflicts with repeated observations of nonlinear concentration–response behaviors that show slower OP_DTT_ increase with the increase of copper (Cu) and manganese (Mn).^5,22–24^

The nonlinearity complicates the interpretation and comparisons of OP_DTT_ across different studies and source apportionment OP. First, measured OP_DTT_ results are highly dependent on experimental conditions, including buffer composition, DTT concentration, and PM_2.5_ loading in the reaction mixture. Second, the lower intrinsic OP_DTT_ values (i.e., mass-normalized OP_DTT_) of PM_2.5_ obtained at higher PM_2.5_ concentrations may reflect a nonlinear assay response rather than real compositional differences. Third, source apportionment approaches based on linear analytical tools, such as multiple linear regression and PMF, may yield false results when OP_DTT_ depends nonlinearly on Cu and Mn concentrations. A mechanistic understanding of this nonlinearity is therefore needed to enable a valid interstudy comparison, interpretation, and prediction of OP_DTT_. To date, however, detailed mechanistic investigations remain limited.

In this work, we measured OP_DTT_ for laboratory-prepared single-metal and mixed-metal solutions and for water extracts of ambient PM_2.5_, and we propose a quasi-Michaelis–Menten mechanism that quantitatively explains the nonlinear response observed in the DTT assay. Because Cu and Mn contribute comparably to OP_DTT_, we further quantify their mixture effect. Results from this work provide mechanistic insight into the nonlinear dependence of OP_DTT_ on PM composition and offer a basis for predicting OP_DTT_ from chemical composition data.

## 2 Materials and Methods

### 2.1 Chemicals and Stock Solutions

The following chemicals and reagents were used in the work: Copper­(II) perchlorate (98%), iron­(II) sulfate heptahydrate (>99%), potassium phosphate monobasic (KH_2_PO_4_), potassium phosphate dibasic (K_2_HPO_4_), imidazole (99%), DTT, and 5,5′-dithio-bis­(2-nitrobenzoic acid) (DTNB) from Sigma-Aldrich; citric acid (99.5%) from Acros Organics; 1,4-naphthoquinone (1,4-NQ) from Fluka; sodium oxalate (99.5%) from AnalaR NORMAPUR; and Chelex 100 resin (100–200 mesh, sodium form) from Bio-Rad. Phosphate buffer (PB, 0.1 M, 77.5 mM K_2_HPO_4_ and 22.5 mM KH_2_PO_4_, pH 7.4) was prepared, treated with Chelex 100 resin to remove trace metals, and stored in a clean, acid-washed (10% HNO_3_ for 24 h) polypropylene bottle. The preparation procedure and effectiveness of Chelex treatment were described in detail in our previous studies.^24,25^

### 2.2 Ambient PM_2.5_ Samples

A total of 62 ambient PM_2.5_ samples were collected on the rooftop of the library at the China University of Petroleum located in Changping District, Beijing, China (40°13′7″N, 116°14′49″E). Sampling was conducted every 6 days from 2 April 2016 to 27 April 2017, and each sampling period lasted 24 h (midnight to midnight). Samples were collected onto prebaked quartz filters using a high-volume aerosol sampler (TH-1000 series, Tianhong Corp., China), operated at a flow rate of 1.13 m^3^/min. After sampling, filters were wrapped in prebaked aluminum foil, transported with ice packs, and stored at −18 °C until analysis.

Filter portions (20 cm^2^) were extracted with 20 mL ultrapure water in an ultrasonic bath for 60 min and filtered through a 0.45 μm PTFE filter. The filtrates were used for analysis of water-soluble organic carbon (WSOC), water-soluble Cu and Mn (WS-Cu and WS-Mn), humic-like substance (HULIS), and OP_DTT_ measurements. Analytical methods of carbonaceous fractions, water-soluble ions, metals, and HULIS are reported in Supporting Section S1 and in our previous publications.^7,23,26^

### 2.3 OP Determination by the DTT Assay

The DTT assay used in this study followed the simplified Cho method introduced in our previous studies.^23,24,27^ Briefly, 50 μL of 2.5 mM DTT was thoroughly mixed with a 200 μL water-extracted PM sample or standard solution in 1 mL PB solution and then incubated at 37 °C for designated incubation times. To guarantee a constant DTT loss rate during the experiment, incubation times ranging from 0 to 90 min were applied such that the total DTT consumption did not exceed around 80–90%.^7,23^ After incubation, 50 μL of 10 mM DTNB were added to quench the remaining DTT. The resulting 2-nitro-5-thiobenzoic acid (TNB) was measured at 412 nm using a PerkinElmer Lambda 950 UV–vis spectrometer within 30 min. Blanks and samples were all measured in triplicate.

The amount of DTT consumed at incubation time *t*, denoted as ΔDTT_*t*_ (μM), is calculated using eq 1:

1 $$\Delta {\mathrm{DTT}}_{t}=\frac{A_{0}-A_{t}}{A_{0}}C_{DTT}$$

where *A*_0_ is the absorbance of the blank at *t* = 0, *A*_*t*_ is the absorbance of the sample at time *t* (min), and *C*_DTT_ is the initial DTT concentration in the reaction solution (100 μM in this study). OP_DTT_ is represented by the DTT loss rate (μM DTT/min), obtained from the slope of ΔDTT_*t*_ versus reaction time *t*. A process blank was performed on every experiment day using the same procedure. The average OP_DTT_ of the process blank was 0.05 ± 0.03 μM/min (*n* = 18). Reported OP_DTT_ values for both ambient samples and lab-prepared solutions were corrected by subtracting the daily process blank.

## 3 Results and Discussion

### 3.1 Nonlinear Relationship between OP_DTT_ and PM_2.5_ Concentrations

We measured OP_DTT_ for 62 ambient PM_2.5_ samples collected in suburban Beijing from April 2016 to April 2017 (Figure 1a). Chemical composition varied substantially across the samples, as seen in Figure 1b and Table S1 (Supporting Information SI). PM_2.5_ concentrations ranged from 8.1 to 192.3 μg/m^3^, and water-soluble Cu ranged from 0.7 to 12.0 ng/m^3^. On average, the water-soluble fractions of these samples produced a DTT loss rate of 0.96 ± 0.41 nmol/min/m^3^. Figure 1a shows OP_DTTv_ as a function of PM_2.5_ air concentration in μg/m^3^, and the PM_2.5_ mass used in the assay is also displayed. The set of 62 data shows a clear nonlinear relationship between the measured OP_DTTv_ and PM_2.5_ concentration. Similar nonlinear behavior has been commonly observed in previous studies^14,15,28–32^ that measured the OP_DTT_ of varied samples (Figure S1 in SI).

**Figure 1:**
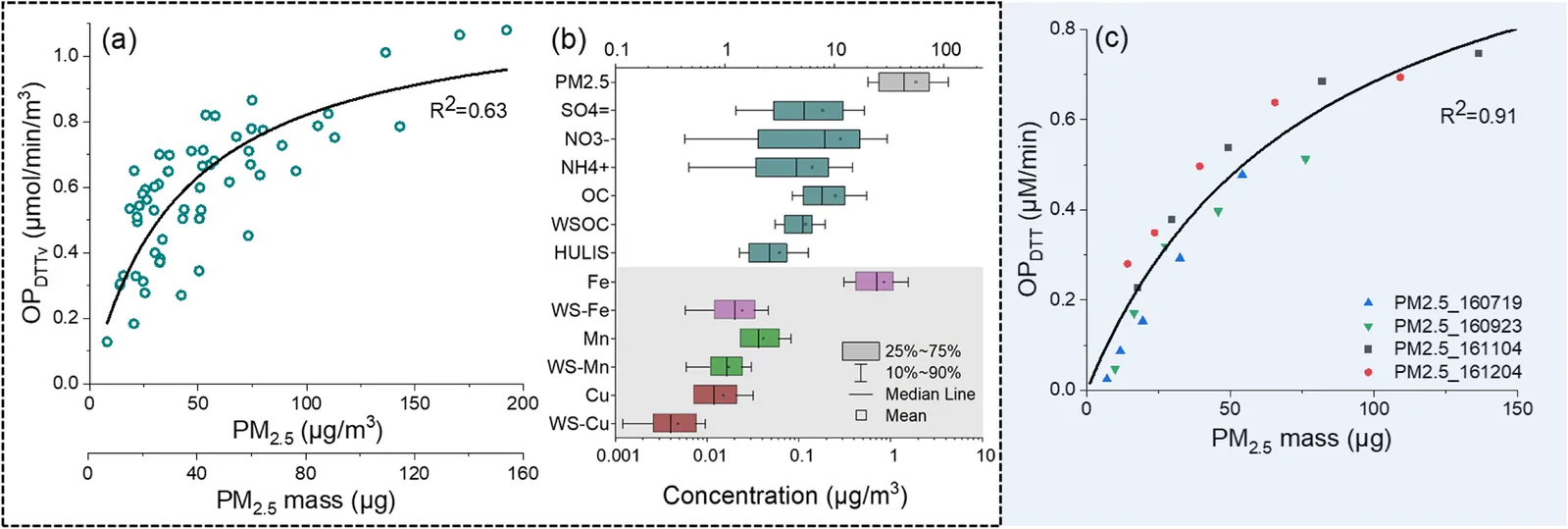
Ambient PM_2.5_ samples show a nonlinear relationship between OP_DTT_ and PM loading. (a) Relationship between OP_DTT_ and PM_2.5_ mass concentration for ambient samples collected in suburban Beijing, China, from April 2016 to April 2017 (*n* = 62); the corresponding PM_2.5_ mass loaded into the assay is also shown. (b) Distribution statistics of PM_2.5_ concentration and selected chemical components in the ambient samples, including water-soluble Mn, Fe, and Cu; OC; and humic-like substances (HULIS). (c) Relationship between OP_DTT_ and PM_2.5_ mass for 20 data points from four ambient PM_2.5_ samples. For each sample, five data points are shown, including the undiluted sample and four successive dilutions; colors indicate different samples.

To further examine the nonlinear behavior, we successively diluted four ambient samples with distinct chemical characteristics (sample IDs: 160719, 160923, 161104, 161204, named according to collection dates in the format of yymmdd) (Table S2 in SI). For instance, Sample 160719 had PM_2.5_ at 67.7 μg/m^3^ and WSOC at 3.0 μg/m^3^, whereas Sample 161104 had a significantly higher PM_2.5_ at 170.7 μg/m^3^ and WSOC at 11.2 μg/m^3^. Each sample was diluted four times, yielding five data points per sample and 20 data points in total. Despite the substantial differences in composition among these samples, all data points aligned along a common nonlinear curve (Figure 1c). This pattern is likely related to the strong correlations of water-soluble Cu and Mn with PM_2.5_ concentration in the Beijing data set (Pearson’s *r* = 0.70 and 0.69, respectively).

These results show that while the nonlinear response is an inherent chemical characteristic of transition metal catalysis, it introduces an operational bias when reporting intrinsic OP_DTTm_ (i.e., mass-normalized OP_DTT_). Because the assay response flattens at higher metal concentrations, higher PM loadings yield systematically lower calculated OP_DTTm_ values. For instance, in Sample 161104, the calculated intrinsic OP_DTTm_ increased from 6.83 pmol/min per μg PM_2.5_ at zero dilution to 15.8 pmol/min per μg PM_2.5_ at an eightfold dilution. Thus, apparent differences in OP_DTTm_ between samples—or even within identical compositions—may simply reflect variations in assay mass loading or dilution rather than genuine differences in toxicological potency, an artifact consistent with the findings of Charrier et al.^22^

### 3.2 Quasi-Michaelis–Menten Mechanism for Nonlinear OP_DTT_ of Cu and Mn

Previous studies have shown that a large fraction of OP_DTT_ in ambient samples can be attributed to transition metals, especially Cu and Mn.^5,33,34^ We therefore hypothesize that the observed nonlinear concentration–response relationship is largely a result of the nonlinear dependence of OP_DTT_ on Cu and Mn concentrations.^5,22–24,35^ The shape of the nonlinear curve in Figure 1c suggests a concentration–response relationship analogous to that described by the Michaelis–Menten (MM) mechanism (Figure 2).

**Figure 2:**
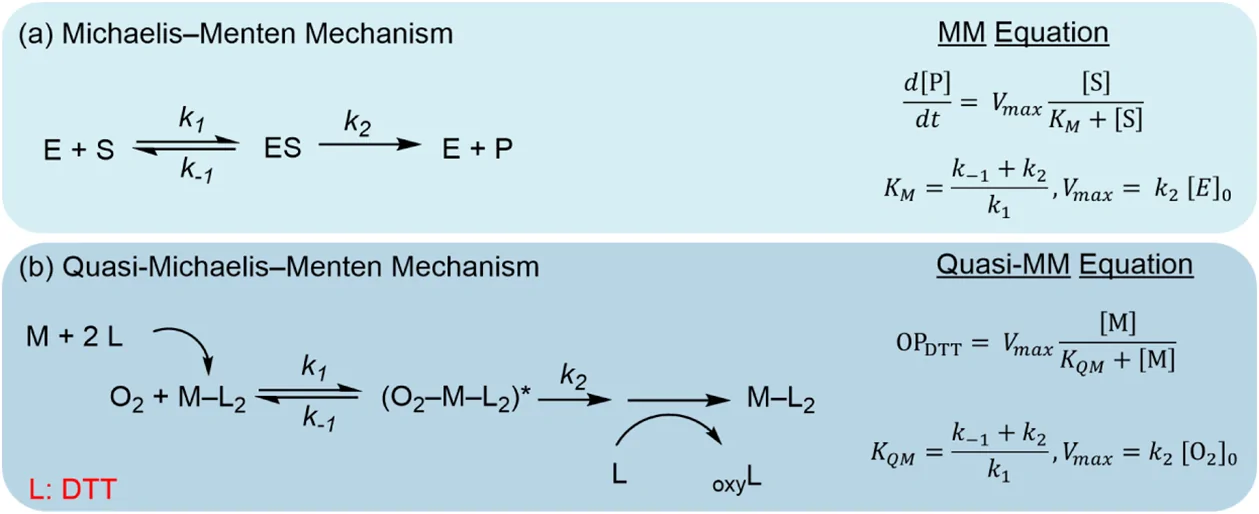
Comparison of the classical Michaelis–Menten (MM) mechanism with the quasi-Michaelis–Menten (quasi-MM) framework proposed here for metal-catalyzed DTT oxidation. (a) Schematic diagram and equations for the MM mechanism of enzyme catalysis, where E is the free enzyme, S is the substrate, ES is the enzyme–substrate complex, P is the reaction product, E_0_ is the total enzyme concentration, *V_max_* is the maximum enzymatic reaction velocity, *K_M_* is the Michaelis–Menten constant, and *k*_1_, *k_–1_, k*_2_ are rate constants for the forward, reverse, and catalytic steps, respectively. (b) Schematic diagram and equations for the quasi-MM mechanism proposed for the consumption of reductants (e.g., DTT) catalyzed by transition metal ions (M), where M-L_2_ represents the metal-DTT complex, (O_2_-M-L_2_)* is the reactive oxygen–metal-DTT intermediate, [O_2_]_0_ is the total dissolved oxygen, *V_max_* is the maximum DTT depletion rate, and *K_QM_* is the quasi-Michaelis–Menten constant.

In biochemistry, the MM mechanism represents the simplest model of enzyme kinetics for the enzyme-catalyzed transformation of a substrate into one product. For the reaction between enzyme (E) and substrate (S) (Figure 2a), the product (P) formation rate is given by eq 2:

2 $$\frac{d\left[P\right]}{dt}=V_{max}\frac{\left[S\right]}{K_{M}+\left[S\right]}$$

3 $$V_{max}=k_{2}{[E]}_{0}$$

4 $$K_{M}=\frac{k_{-1}+k_{2}}{k_{1}}$$

where [S] is the substrate concentration, [E]_0_ is the total enzyme concentration, *V*_*max*_ denotes the maximum reaction velocity when all the enzyme is complexed with the substrate, and *K*_*M*_ is the pseudo-equilibrium constant called the Michaelis–Menten constant.^36^ By analogy, a quasi-MM mechanism is proposed to describe the reactions among DTT, Cu (or Mn), and O_2_ (Figure 2b). A stepwise analogy is shown in Section S2 in SI. A more mechanistically explicit scheme is shown in Figure 3.

**Figure 3:**
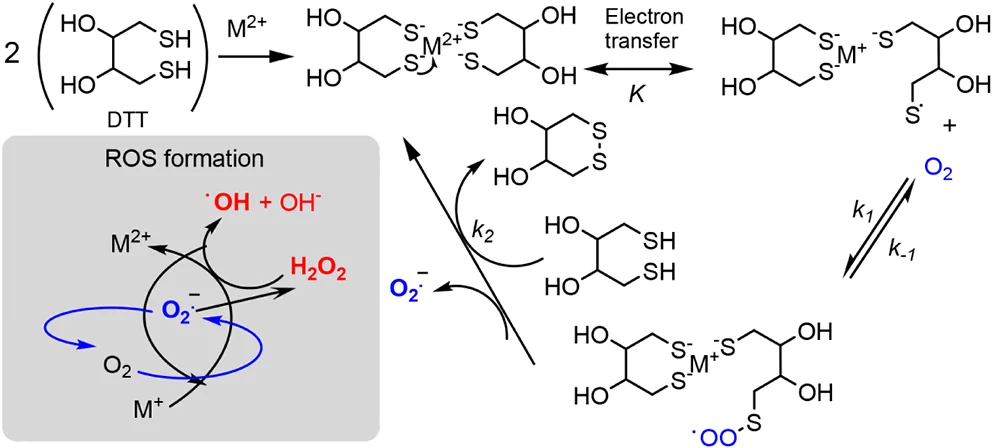
Proposed reaction scheme of metal ion (M^2+^)-catalyzed DTT oxidation and generation of •OH and other ROS (adapted from *Kachur et al*.)^35^

DTT contains two sulfhydryl groups and two hydroxyl groups, which give it strong metal-coordinating ability. Notably, no free metal ions such as Zn­(II), Pb­(II), Cd­(II), Cu­(I), or Ni­(II) have been reported in the presence of DTT at pH 7.4.^37^ We therefore infer that the dominant Cu-containing species in the Cu–DTT reaction system is the Cu–DTT complex.^24,35^ Under our assay conditions, Cu or Mn is expected to coordinate rapidly with DTT because DTT has strong binding ability and is present at a much higher concentration than the metals (see eqs S6, S15, and S16). In this case, the major species in solution are free DTT and M–DTT complexes. We assume that the M–DTT complex is the key reactive intermediate that undergoes intramolecular electron transfer from sulfur to the metal center, for example to form an M^+^–DTT* intermediate.^35^ This intermediate then reacts with dissolved O_2_ to form an unstable (O_2_–M^+^–DTT)* intermediate, which subsequently dissociates or decomposes to oxidized DTT and regenerates the M–DTT complex.

Analogous to the derivation of product formation in the enzyme-catalyzed reaction, the consumption rate of DTT can be written as eq 5, with the two parameters, *V*_*max*_ *and K*_*QM*_, defined in eqs 6 and 7:

5 $${\mathrm{OP}}_{\mathrm{DTT}}=\frac{d\left[\mathrm{DTT}\right]}{dt}=V_{max}\frac{\left[M\right]}{K_{QM}+\left[M\right]}$$

6 $$V_{max}=k_{2}{[O_{2}]}_{0}$$

7 $$K_{QM}=\frac{k_{-1}+k_{2}}{Kk_{1}}$$

where *k*_1_, *k*_*–1*_, and *k*_2_ are reaction rate constants for the elementary steps shown in Figure 3 and *K* is the equilibrium constant for the intramolecular electron-transfer step. The physical meaning of *V*_*max*_ is the maximum DTT loss rate (OP_DTT_) that can be achieved in the reaction system within a certain range of metal concentration (see discussion below), while *K*_*QM*_ is numerically equal to the metal concentration at which OP_DTT_ equals half of *V*_*max*_.

For the two metals considered here, the resulting OP_DTT_ (μM/min) expressions are provided in eqs 8 and 9 (see Section S3 eqs S1–S7 for a detailed derivation in the SI):

8 $${\mathrm{OP}}_{\mathrm{Cu}}(\mathrm{\mu M}/\min)=V_{\max}\frac{{\left[{\mathrm{Cu}}^{2+}\right]}_{0}}{K_{\mathrm{QM}}+{\left[{\mathrm{Cu}}^{2+}\right]}_{0}}$$

9 $${\mathrm{OP}}_{\mathrm{Mn}}(\mathrm{\mu M}/\min)=V_{\max}^{\prime }\frac{{\left[{\mathrm{Mn}}^{2+}\right]}_{0}}{K_{\mathrm{QM}}^{\prime }+{\left[{\mathrm{Mn}}^{2+}\right]}_{0}}$$

where [Cu^2+^]_0_ and [Mn^2+^]_0_ denote the initial concentrations in the reaction system, *V*_*max*_ and *K*_*QM*_ parameters denote those for the Cu–DTT system, whereas *V′*_*max*_ and *K′*_*QM*_ denote those for the Mn–DTT system.

The derived OP_DTT_ equations successfully describe the dependence of OP_DTT_ on the initial metal ion concentration in the laboratory-prepared metal solutions (Figure 4; *R* ^2^ = 0.997). The concentration ranges of Cu (0–0.2 μM) and Mn (0–0.6 μM) correspond to those observed in the ambient samples in this study (Table S1). The two fitted parameters *V*_*max*_ and *K*_*QM*_ are also obtained:

$$\mathrm{Cu-DTT assay:}\;V_{\max}=0.573\pm 0.018\mathrm{\mu M}/\min,,K_{\mathrm{QM}}=0.0402\pm 0.003\mathrm{\mu M}$$

$$\mathrm{Mn-DTT assay:}\;V_{\max}^{\prime }=0.733\pm 0.032\mathrm{\mu M}/\min,,K_{\mathrm{QM}}^{\prime }=0.219\pm 0.022\mathrm{\mu M}$$

**Figure 4:**
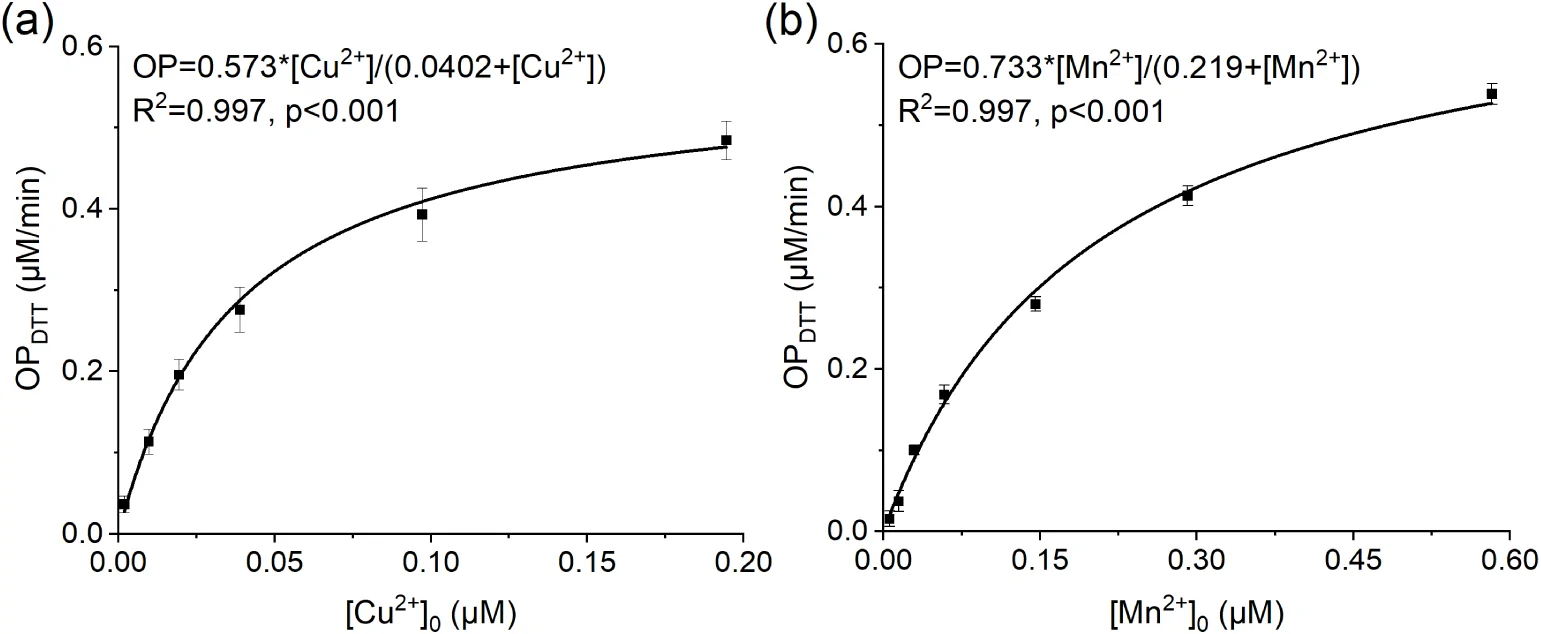
OP_DTT_ exhibits quasi-Michaelis–Menten concentration–response behavior for both Cu^2+^ and Mn^2+^. (a) Concentration–response curve of OP_DTT_ versus Cu^2+^. (b) Concentration–response curve of OP_DTT_ versus Mn^2+^. Black lines show the quasi-MM fits, and black circles with error bars show the mean and standard error of slopes obtained from independent triplicate measurements.

We also successfully applied the quasi-MM fit to concentration–response data reported in a previous study, which had been fitted only with an empirical power function lacking mechanistic interpretation (Figure S2).^5,22^ It should be noted that the parameters in eq 5 (*V*_*max*_ and *K*_*QM*_) may vary among different DTT assay protocols, for example, different DTT concentrations in the reaction system,^23,35^ and may also depend on the metal concentration range used for quasi-MM fitting.

This proposed mechanism also explains why DTT consumption should not exceed about 80–90% if a proportional relationship between DTT consumption and reaction time is to be maintained. At the beginning of the reaction, [DTT]_0_ ≫ [M]_0_, and the [M–DTT] complex concentration is approximately equal to [M]_0_, so OP_DTT_ primarily depends on [M]_0_. As the reaction proceeds and DTT consumption exceeds about 80–90%, however, the condition [DTT] ≫ [M]_0_ may no longer hold. When little DTT remains, i.e., [DTT] ≪ [M]_0_), [M–DTT] ≈ [DTT] ≪ *K*_*QM*_, OP_DTT_ becomes dependent on the remaining DTT concentration rather than being approximately constant (eq 5). Under these conditions, the DTT loss rate becomes *V_max_* [DTT]/*K_QM_*.

### 3.3 Comparison of Underlying Mechanisms for OP_DTT_, OP_AA_, and OP_GSH_ of Cu

The quasi-MM behavior observed for the DTT assay differs from that previously reported for the ascorbic acid (AA)^38^ and glutathione (GSH)^39^ assays. In the AA assay, Cu can oxidize AA through an additional direct pathway, beyond the MM-like pathway. As a result, OP_AA_ is nonlinear at low Cu concentrations but becomes increasingly linear at higher Cu concentrations (Figure 5a). In contrast, the DTT assay lacks a direct oxidation pathway. Consequently, OP_DTT_ increases nearly linearly at low Cu concentrations and approaches a plateau at higher concentrations.

**Figure 5:**
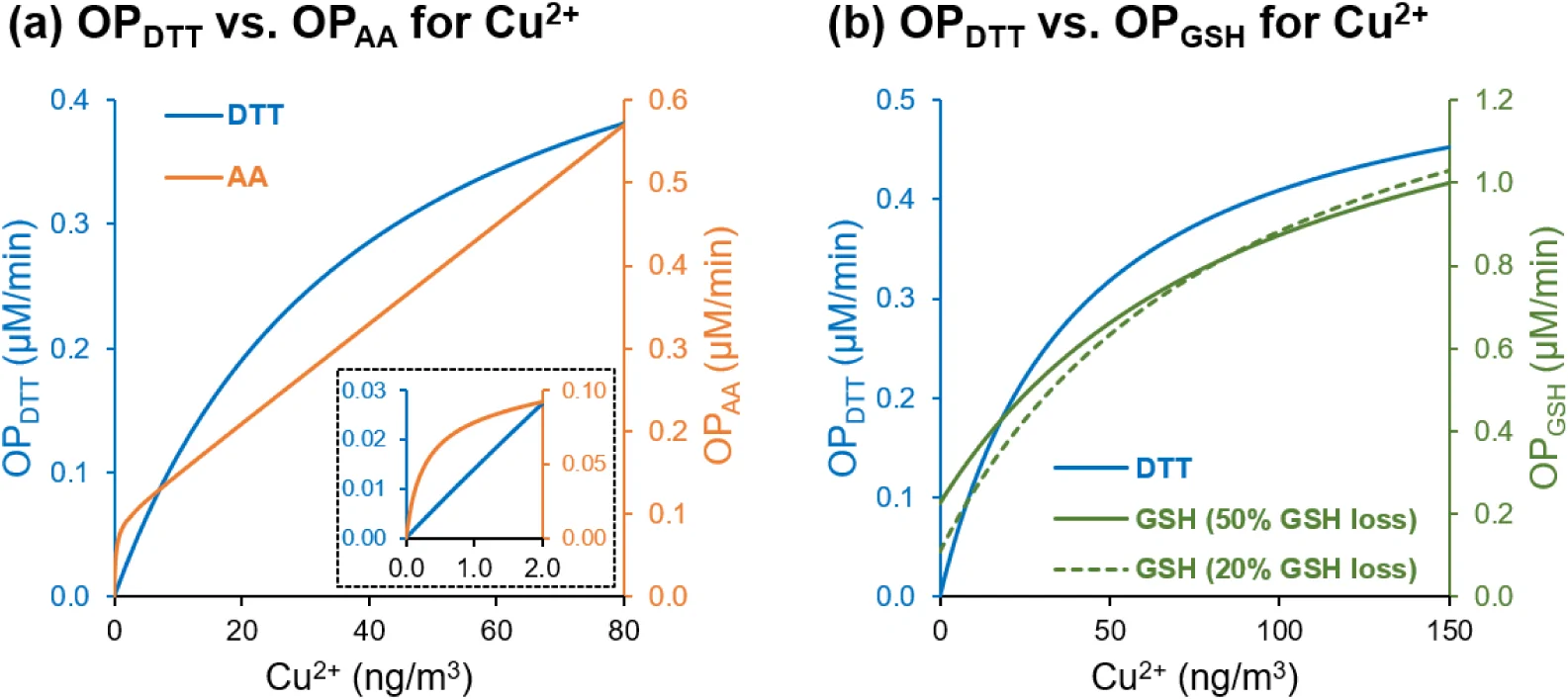
Concentration–response behavior of OP_DTT_ differs from that of the AA and GSH assays for Cu^2+^. (a) OP_DTT_ versus OP_AA_; (b) OP_DTT_ versus OP_GSH_.

The GSH assay shares a similar thiol-based reaction pathway to the DTT assay and lacks an analogous direct oxidation pathway. However, the instantaneous GSH loss rate depends on the remaining GSH concentration in the system, causing the concentration–response curve to evolve over time (Figure 5b). In contrast, under our assay conditions, DTT loss follows apparent zero-order kinetics throughout the reaction period; therefore, a single MM-like curve adequately represents the response. Additionally, because Cu and Mn contribute comparably in the DTT assay, mixed-metal effects are more important for OP_DTT_ than for OP_AA_ or OP_GSH_.

### 3.4 OP_DTT_ of Cu and Mn Mixtures

In our previous work on the AA and GSH assays, the mechanistic analysis focused primarily on Cu because Cu dominates OP in those assays. By contrast, Cu and Mn contribute comparably to OP in the DTT assay (Figure 4), making mixed-metal effects non-negligible. Overall, the underlying mechanism for OP_DTT_ is simpler than those for OP_AA_ ^38^ and OP_GSH_,^39^ which facilitates analysis of transition metal mixture effects in the DTT assay.

In the presence of both Cu and Mn, competition between the two metals is expected during electron transfer from DTT to O_2_. The resulting expression for OP_DTT_ in mixed Cu–Mn systems, OP_Mn&Cu_, is given in eq 10 (see Section S4 eqs S8–S17 for a detailed derivation in the SI):

$${\mathrm{OP}}_{\mathrm{Mn}\&\mathrm{Cu}}\left(\mathrm{\mu M}/\min\right)=\frac{\frac{V_{\max}}{K_{QM}}{\left[{\mathrm{Cu}}^{2+}\right]}_{0}+\frac{V_{max}^{’}}{K_{QM}^{’}}{\left[{\mathrm{Mn}}^{2+}\right]}_{0}}{1+{\left[{\mathrm{Cu}}^{2+}\right]}_{0}/KQM+{\left[{\mathrm{Mn}}^{2+}\right]}_{0}/K_{QM}^{’}}$$ 10

We measured OP_DTT_ for 34 laboratory-prepared Cu–Mn solutions. Their individual compositions are tabulated in Table S3, with Cu^2+^ in the range of 0.007–0.202 μM, Mn^2+^ in the range of 0.001–0.223 μM, and the concentration ratios of [Cu^2+^]/[Mn^2+^] in the range of 0.433–8.82. Figure 6 compares the measured OP_Mn&Cu_ and those predicted using eq 10. A near unity slope (0.99) and a Pearson’s *r* of 0.986 are obtained, supporting the validity of eq 10.

**Figure 6:**
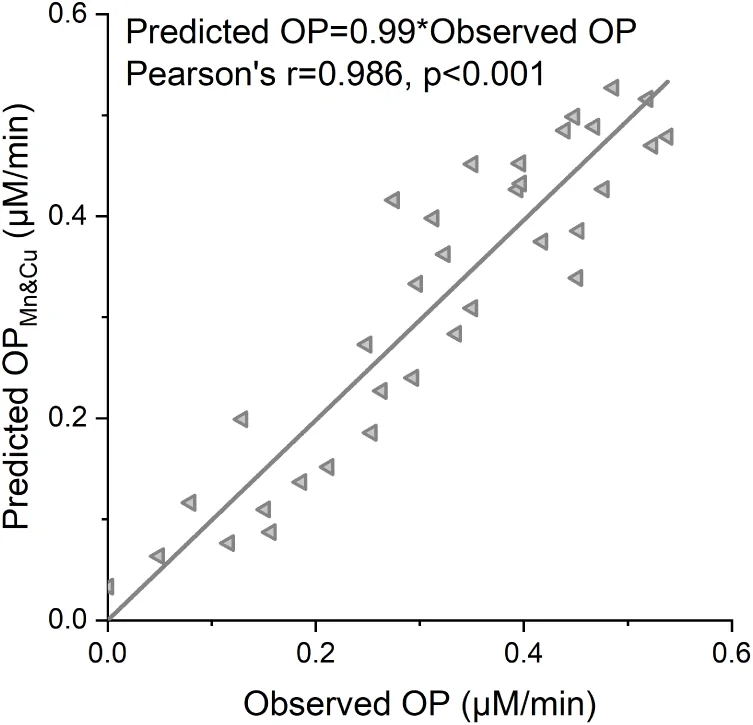
Predicted OP_Mn&Cu_, including mixture effects, versus measured OP for laboratory-prepared Cu–Mn mixed metal solutions (Mn and Cu, *n* = 34).

Equation 10 allows us to quantify mixing effects between the two coexisting metal ions. We define an index of mixture effect (*I*_*ME*_) as the ratio of the DTT loss rate of the mixture to the arithmetic sum of the DTT loss rates of the individual components,^23,24^ as shown in eq 11:

11 $$I_{ME}=\frac{{\mathrm{OP}}_{\mathrm{DTT},mix}}{\underset{i}{\sum }{\mathrm{OP}}_{\mathrm{DTT},i}}$$

*I*_*ME*_ describes the magnitude and direction of the mixture effect. An *I*_*ME*_ of 1 indicates no mixture effect. *I*_*ME*_ > 1 indicates enhancement (synergy), and *I*_*ME*_ < 1 indicates suppression (antagonism). The theoretical *I*_*ME*_ values calculated from eqs 8–10 (eq S18) show that Cu–Mn mixtures are consistently antagonistic (*I*_*ME*_ < 1), consistent with the proposed competition mechanism (Figure S3).

### 3.5 Predicting OP_DTT_ of Ambient Samples

Figure 7a shows the response surface predicted from Cu and Mn concentrations, together with measured OP_DTT_ for ambient samples. On average, OP_Mn&Cu_ calculated from eq 10 accounts for 68 ± 11% of the measured OP_DTT_ for the ambient samples. In addition to redox-active transition metals, redox-active organics also contribute to OP in ambient PM.^5,40^ Assuming that contributions from other metals are minor,^5^ the residual term, ΔOP (measured OP minus predicted OP_Mn&Cu_), can be approximated as the contribution from organic compounds. This interpretation is supported by the strong correlation between ΔOP and WSOC (Figure 7b; Pearson’s *r* = 0.94, *p* < 0.001).

**Figure 7:**
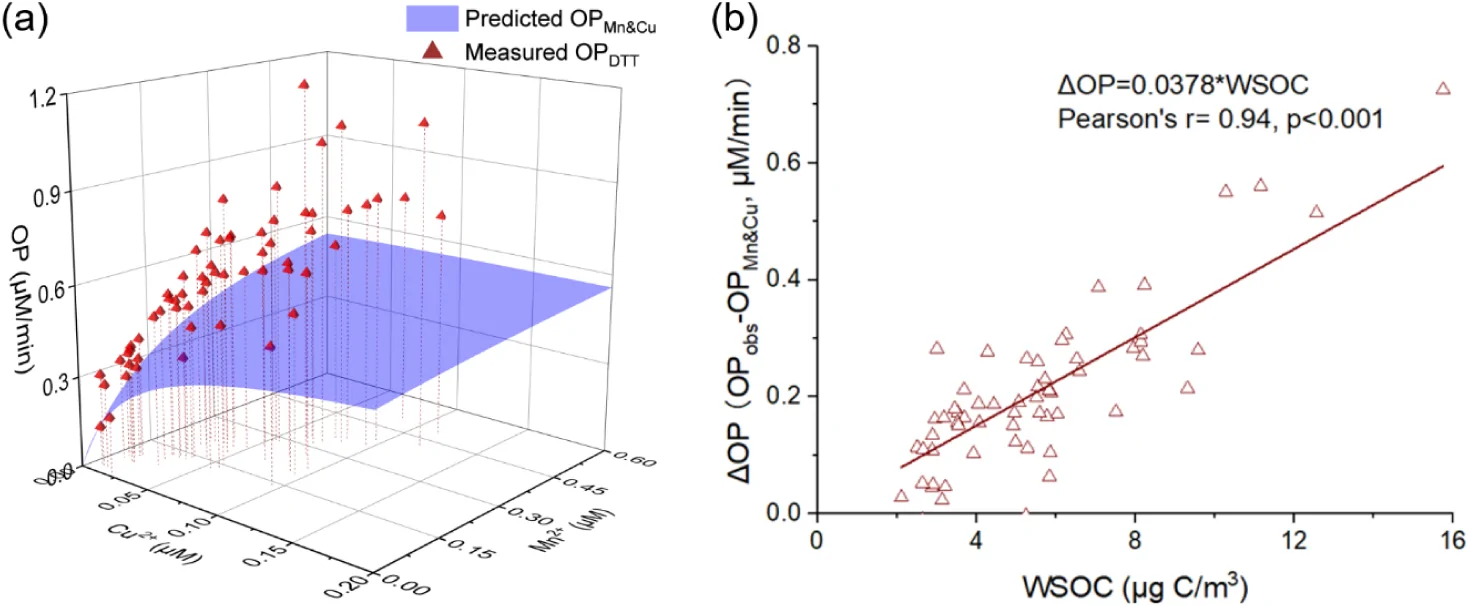
Cu–Mn mixing effect is consistently antagonistic, and Cu, Mn, and WSOC together explain most of the variability in OP_DTT_ for ambient PM_2.5_ samples. (a) Three-dimensional response surface of OP_DTT_ predicted from Cu and Mn concentrations using eq 10, together with measured OP_DTT_ values for ambient samples (*n* = 62). (b) Relationship between ΔOP (OP_obs_ – OP_Mn&Cu_) and WSOC in ambient samples.

The effects of organics on OP in the DTT assay may arise from three possible mechanisms: (1) direct contribution to OP by redox-active organics; (2) modification of M–DTT redox activity through complexation; and (3) changes in the solubility of TMs through complexation.^7,24,25,41–43^ Because this study focuses on the OP_DTT_ of water-soluble PM_2.5_, the solubility effect is already incorporated during the water extraction. ΔOP therefore includes only contributions from mechanisms 1 and 2.

To assess the relative importance of these two mechanisms in ambient samples, we conducted additional experiments using oxalate, citric acid, and imidazole, which represent common ligands in PM_2.5_ (mechanism 2), and 1,4-NQ, which represents a redox-active organic compound (mechanism 1) (Figures S4–S6). The results indicate that the influence of ligand-induced modification of TM activity is negligible relative to the direct contribution of redox-active organics under our experimental conditions (as shown in Figures S4 and S5). Charrier et al.^5^ also reported a linear concentration–response relationship for quinones in the DTT assay. We therefore approximate the organic contribution to OP_DTT_ as linearly proportional to WSOC:

12 $${\mathrm{OP}}_{\mathrm{orgs}}=\underset{i}{\sum }\beta_{i}{[\mathrm{Org}]}_{i}=\underset{i}{\sum }\alpha_{i}\beta_{i}\left[\mathrm{WSOC}\right]$$

where α_*i*_ represents the fraction of redox-active compounds in WSOC (%) and β_*i*_ is the concentration–response factor for these compounds. We note that the value of $\underset{i}{\sum }\alpha_{i}\beta_{i}$ may vary among samples due to regional differences in WSOC sources, composition of redox-active organics (e.g., quinones vs aliphatic compounds), and degree of atmospheric oxidation.^40^ While the overall model structure combining nonlinear metal kinetics with a linear WSOC term is transferable across ambient environments, the empirical value of $\underset{i}{\sum }\alpha_{i}\beta_{i}$ reflects local WSOC characteristics and should be determined locally when applying this framework to different geographic regions. Because of the limited data set and the strong linear correlation observed in Figure 7b, we use the slope of that correlation as an empirical estimate of $\underset{i}{\sum }\alpha_{i}\beta_{i}$ . Since the primary focus of this work is the quantitative relationship between transition metals and OP_DTT_, further investigation of organic contributions is beyond the scope of this study.

The OP of water-soluble PM_2.5_ can then be estimated from the concentrations of Cu, Mn, and WSOC using eq 13:

$$\mathrm{OP}\left(\mathrm{\mu M}/\min\right)=\frac{\frac{V_{\max}}{K_{QM}}{\left[{\mathrm{Cu}}^{2+}\right]}_{0}+\frac{V_{max}^{’}}{K_{QM}^{’}}{\left[{\mathrm{Mn}}^{2+}\right]}_{0}}{1+{\left[{\mathrm{Cu}}^{2+}\right]}_{0}/K_{QM}+{\left[{\mathrm{Mn}}^{2+}\right]}_{0}/K_{QM}^{’}}+\underset{i}{\sum }\alpha_{i}\beta_{i}\left[\mathrm{WSOC}\right]$$ 13

Applying this equation to the 62 ambient samples, this model accounts for 98.6% of the measured OP_DTT_ (Figure 8), supporting the proposed framework.

**Figure 8:**
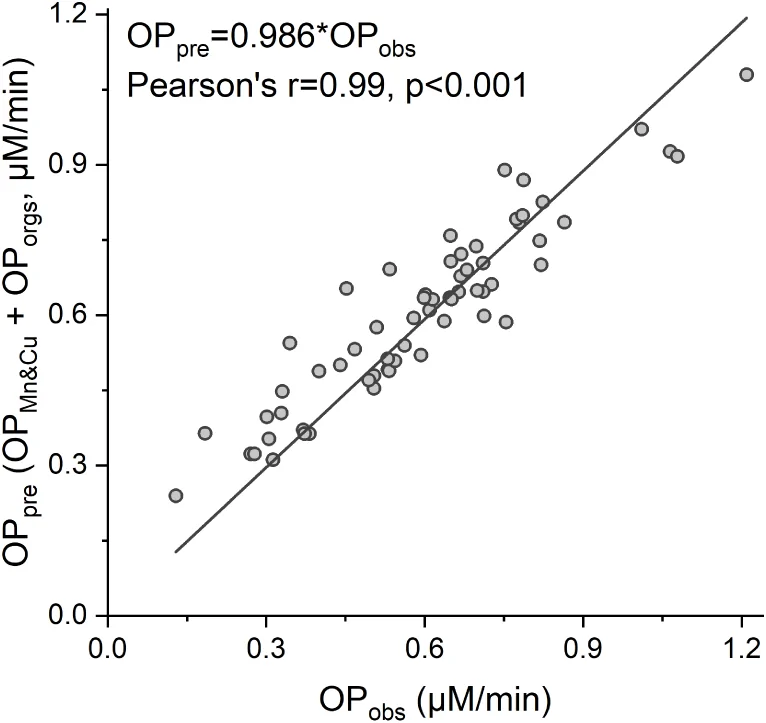
OP values predicted using eq 13 versus measured OP values for 62 ambient PM_2.5_ samples collected in Beijing over a year.

## 4 Implications

This study proposes a plausible mechanism for reactions among DTT, transition metals (Cu and Mn), and O_2_ in the DTT assay. The resulting quasi-MM framework quantitatively captures the nonlinear relationship between the metal concentration and OP_DTT_ for Cu, Mn, and their mixtures. It also explains the antagonistic interaction between Cu and Mn and, with a simple WSOC term, provides an effective basis for predicting OP_DTT_ from the PM chemical composition. Mechanistically, this study clarifies why the intrinsic OP_DTT_ can exhibit mass-dependent artifacts. This finding has important implications for comparing the intrinsic OP_DTT_ across studies. The proposed equations may provide a basis for normalizing or correcting OP_DTT_ measured under different PM-loading conditions, thereby improving comparability.

In addition to nonlinear saturation kinetics, metal precipitation represents another critical source of analytical bias in acellular OP assays.^44^ At near-neutral pH (e.g., pH 7.4 phosphate buffer used in the DTT assay), transition metals such as Fe^3+^ or high concentrations of Cu^2+^/Mn^2+^ can undergo hydrolysis or phosphate precipitation, thereby reducing the dissolved bioavailable metal concentration during incubation. This solubility constraint can cause an underestimation of metal-driven OP_DTT_ at high PM loadings, further compounding the nonlinear mass-dependent artifacts observed in the experimental protocols.

The results also have broader implications for interpreting the oxidative potential across different acellular assays. Because the DTT, AA, and GSH assays involve different reaction pathways and concentration–response behaviors, they do not necessarily yield identical OP values for the same PM sample or chemical component. As discussed in Section 3.3, OP_AA_ includes an additional direct oxidation pathway, whereas OP_DTT_ follows a quasi-MM-type response, and OP_GSH_ depends more explicitly on the remaining antioxidant concentration during the reaction. Accordingly, differences among assay-specific OP values should be interpreted as reflecting differences in chemical sensitivity and mechanism rather than analytical inconsistency alone. This distinction is important when comparing results across studies or when selecting an assay to represent specific aspects of the PM redox activity.

Our results also emphasize the importance of using a trace-metal-free buffer. Because OP_DTT_ responds nonlinearly to metal concentrations, metal contamination in the buffer can interact with metals in the sample and bias the measured response. In such cases, simple blank subtraction may not fully correct the artifact and may lead to an underestimation of sample OP_DTT_.

Finally, similar nonlinear metal concentration–response behavior has been reported in other acellular assays targeting specific ROS, including the hydroxyl radical and hydrogen peroxide.^45,46^ The quasi-MM framework developed here may also be useful for investigating the kinetics of these related systems.

## Supplementary Material



## Notes

Analytical details for chemical characterizations, including carbonaceous fractions, water-soluble ions, metals, and HULIS (Section S1); derivations of quasi-Michaelis–Menten rate expressions for single and mixed metal systems (Sections S2–S4); summary chemical composition data and dithiothreitol oxidative potential (OP_DTT_) for ambient and simulated samples (Tables S1–S3); comparison of OP_DTT_ responses across ambient studies, metal mixtures, and organic additives (Figures S1–S6) (PDF)
