# Supplementary material for: A Quasi-Michaelis–Menten Mechanism Explains the Nonlinear Relationship between Transition Metals and PM2.5 Oxidative Potential Measured by the Dithiothreitol (DTT) Assay
Source: Environ Sci Technol. 2026 Sep 19;60(39):27640–8. doi: 10.1021/acs.est.6c09293 (PMC13648234; doi:10.1021/acs.est.6c09293)
Supplement: Supplementary file 1 [file es6c09293_si_001.pdf]

Supplementary Information for

A Quasi-Michaelis–Menten Mechanism Explains the Nonlinear Relationship between Transition Metals and $PM_{2.5}$ Oxidative Potential Measured by the Dithiothreitol (DTT) Assay

Hanzhe Chen[1], Yuhuang Cheng[2], Kezheng Liao[2], Manfei Lin[3], Tao Li[1,4], Jian Zhen Yu[1,2,*]

[1]Division of Environment and Sustainability, tThe Hong Kong University of Science and Technology, Hong Kong 999077, China

[2]Department of Chemistry, The Hong Kong University of Science and Technology, Hong Kong 999077, China

[3]Institution of Atmospheric Environment, Jiangsu Provincial Academy of Environmental Science, Nanjing, Jiangsu 210036, China

[4]School of Environmental Science and Engineering, Shandong University, Qingdao, Shandong 266237, China

*Corresponding author. Email: chjianyu@ust.hk

This document contains 4 supporting sessions, 3 tables and 6 figures.

Section S1. Analytical Details for Chemical Characterization

S1.1. Carbonaceous Fractions

Water-Soluble Organic Carbon (WSOC): WSOC concentrations in the aqueous extracts were quantified using a Total Organic Carbon Analyzer (TOC-VCPH, Shimadzu, Japan). Potassium hydrogen phthalate (KHP) was used to construct 5-point calibration curves (0.0–10.0 mg C L^{-1}, $R^2 > 0.99$). Each sample was injected in triplicate, yielding a coefficient of variation (CV) $< 3\%$.

Organic Carbon (OC) and Elemental Carbon (EC): Quartz filter punches were analyzed for OC and EC using the thermal-optical reflectance (TOR) method following the IMPROVE_A protocol on a DRI Model 2001 Thermal/Optical Carbon Analyzer (Desert Research Institute, USA).

S1.2. Water-Soluble Inorganic Ions

Major water-soluble inorganic ions (NH_4^+, SO_4^{2-}, NO_3^-, Cl^-) in the aqueous extracts were determined using an Ion Chromatograph (Dionex ICS-1100, Thermo Scientific, USA). Multi-ion standard solutions (0.0–10.0 mg L^{-1}, $R^2 > 0.99$) were used for multi-point calibration, and field blanks were processed in parallel to perform background subtraction.

S1.3. Trace Metals

Water-Soluble Metals: Concentrations of water-soluble metals in the aqueous extracts were measured using an Inductively Coupled Plasma Optical Emission Spectrometer (ICP-OES, Avio 200, PerkinElmer, USA). Multi-element standard solutions were used for calibration (0.0–100.0 μg L^{-1}, $R^2 > 0.99$).

Total Metals: Total metal concentrations were determined using quartz filter punches subjected to acid digestion (HNO_3/HCl), followed by analysis on the same ICP-OES instrument under identical calibration conditions.

S1.4. Humic-like Substances (HULIS)

HULIS-C was isolated from aqueous PM extracts using Oasis HLB cartridges (Waters, USA) according to established protocols. Briefly, the aqueous extract was loaded onto a pre-conditioned HLB cartridge. Hydrophilic organic species and inorganic ions were removed by washing with ultrapure water, and the hydrophobic HULIS fraction was eluted with methanol.

The eluted fraction was evaporated to dryness under a gentle stream of high-purity N_2 gas, reconstituted in ultrapure water, and analyzed for HULIS concentration via evaporative light-scattering detector (ELSD3300, Alltech, USA).

Section S2. Step-wise analogy of Michaelis–Menten (MM) Mechanism for enzyme catalysis and quasi-MM mechanism for reactions underlying OP_{DTT}

	Michaelis–Menten (MM) Mechanism for Enzyme Catalysis	Pseudo-MM Mechanism for OP measured as reductant consumption
	$$E + S \underset{k_{-1}}{\overset{k_1}{\rightleftharpoons}} ES \xrightarrow{k_2} P$$	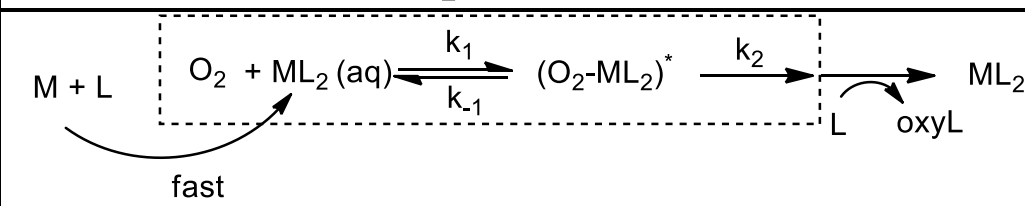 L: DTT or other reductant capable of chelating with M ions.
Step 1	Product formation rate: $$\frac{d[\mathrm{P}]}{dt} = k_2[\mathrm{ES}]$$	DTT consumption rate: $$OP_{DTT} = \frac{-d[\mathrm{DTT}]}{dt} = k_2[(\mathrm{O_2ML_2})^*]$$
Step 2	Pseudo-state approximation for ES: $$k_1[\mathrm{E}][S] = k_{-1}[\mathrm{ES}] + k_2[\mathrm{ES}]$$	Pseudo-state approximation for $(O_2ML_2)^*$: $$k_1[\mathrm{O_2}]_{aq}[\mathrm{ML_2}] = (k_{-1} + k_2)[(\mathrm{O_2ML_2})^*]$$
Step 3	The total amount of E: $$[\mathrm{E}]_0=[\mathrm{E}]+[\mathrm{ES}]$$	The total amount of $[O_2]$: $$[\mathrm{O_2}]_0=[\mathrm{O_2}]_{aq}+[(\mathrm{O_2ML_2})^*]$$
Step 4	Solving this for [E] and substituting this result in PSS for [ES]: $$[\mathrm{ES}] = \frac{k_1[\mathrm{S}]([\mathrm{E}]_0 - [\mathrm{ES}])}{k_{-1} + k_2}$$	Solving this for $[(O_2ML_2)^*]$ and substituting this result in PSS for [ES]: $$[(\mathrm{O_2ML_2})^*] = \frac{k_1[\mathrm{ML_2}]([\mathrm{O_2}]_0 - [(\mathrm{O_2ML_2})^*])}{k_{-1} + k_2}$$
Step 5	Solving this for [ES]: $$[\mathrm{ES}] = \frac{k_1[\mathrm{S}][\mathrm{E}]_0}{(k_{-1} + k_2) + k_1[\mathrm{S}]}$$	Solving this for $[(O_2ML_2)^*]$: $$[(\mathrm{O_2ML_2})^*] = \frac{k_1[\mathrm{ML_2}][\mathrm{O_2}]_0}{(k_{-1} + k_2) + k_1[\mathrm{ML_2}]}$$
Step 6	Substituting Eq. (5) into Eq. (1): $$\frac{d[\mathrm{P}]}{dt} = \frac{k_1k_2[\mathrm{S}][\mathrm{E}]_0}{(k_{-1} + k_2) + k_1[\mathrm{S}]}$$	Substituting Eq. (5) into Eq. (1): $$OP_{DTT} = \frac{k_1k_2[\mathrm{ML_2}][\mathrm{O_2}]_0}{(k_{-1} + k_2) + k_1[\mathrm{ML_2}]}$$
Step 7	Setting (a) Michaelis-Menten constant $K_M = (k_{-1}+k_2)/k_1$, and (b) $V_{max} = k_2[E]_0$, we get MM equation: $$\frac{d[\mathrm{P}]}{dt} = V_{max}\frac{[\mathrm{S}]}{K_M + [\mathrm{S}]}$$	Setting (a) Pseudo M-M constant K_{QM}’ $=(k_{-1}+k_2)/k_1$, and (b) $V_{max} = k_2[O_2]_0$, we get the pseudo-MM equation: $$OP_{DTT} = V_{max}\frac{[\mathrm{ML_2}]}{K_{QM} + [\mathrm{ML_2}]}$$
		Since $[ML_2] \approx [M]_0$, Eq (7a) becomes: $$OP_{DTT} = V_{max}\frac{[\mathrm{M}]_0}{K_{QM} + [\mathrm{M}]_0}$$

Section S3: Chemical reactions for single metal system (Cu):

$$Cu^{2+} + DTT \xrightarrow{\text{fast}} Cu^{2+}\text{–}DTT \underset{K}{\overset{\text{electron transfer}}{\longleftrightarrow}} Cu^{+}\text{–}DTT^{*} + O_2 \underset{k_{-1}}{\overset{k_1}{\rightleftharpoons}} Cu^{+}\text{–}DTT\text{–}O_2^{*} \xrightarrow{k_2} Cu^{2+}\text{–}DTT + oxyDTT$$

Equilibrium:

$$[O_2]+[Cu^{+}\text{–}DTT\text{–}O_2^{*}] = [O_2]_0 \quad (S1)$$

$$k_1[Cu^{+}\text{–}DTT^{*}][O_2] = k_{-1}[Cu^{+}\text{–}DTT\text{–}O_2^{*}]+k_2[Cu^{+}\text{–}DTT\text{–}O_2^{*}] \quad (S2)$$

$$K[Cu^{2+}\text{–}DTT] = [Cu^{+}\text{–}DTT^{*}] \quad (S3)$$

Derivation:

$$k_1[Cu^{+}\text{–}DTT^{*}]([O_2]_0\text{-}[Cu^{+}\text{–}DTT\text{–}O_2^{*}]) = (k_{-1}+k_2)[Cu^{+}\text{–}DTT\text{–}O_2^{*}] \quad (S4)$$

$$[Cu^{+}\text{–}DTT\text{–}O_2^{*}] = \frac{[O_2]_0[Cu^{+}\text{–}DTT^{*}]}{\frac{k_{-1}+k_2}{k_1}+[Cu^{+}\text{–}DTT^{*}]} = \frac{[O_2]_0[Cu^{2+}\text{–}DTT]}{\frac{k_{-1}+k_2}{Kk_1}+[Cu^{2+}\text{–}DTT]} \quad (S4)'$$

$$OP_{DTT}\ (\mu M/min) = \frac{-d[DTT]}{dt} = \frac{d[oxyDTT]}{dt} = k_2[Cu^{+}\text{–}DTT\text{–}O_2^{*}] = k_2[O_2]_0\frac{[Cu^{2+}\text{–}DTT]}{\frac{k_{-1}+k_2}{Kk_1}+[Cu^{2+}\text{–}DTT]}$$

$$= V_{max}\frac{[Cu^{2+}\text{–}DTT]}{K_{QM}+[Cu^{2+}\text{–}DTT]} \qquad \left(k_2[O_2]_0 = V_{max};\ K_{QM} = \frac{k_{-1}+k_2}{Kk_1}\right) \quad (S5)$$

Approximation:

$$[Cu^{2+}\text{–}DTT]\approx[Cu^{2+}]_0\ ([DTT]_0\gg[Cu]_0) \quad (S6)$$

Result:

$$OP_{DTT\text{-}Cu}\ (\mu M/min) = V_{max}\frac{[Cu^{2+}]_0}{K_{QM}+[Cu^{2+}]_0} \quad (S7)$$

Section S4: Chemical reactions for mix metals system (Cu-Mn):

$$Cu^{2+} + DTT \xrightarrow{\text{fast}} Cu^{2+}\text{–}DTT \underset{K}{\overset{\text{electron transfer}}{\longleftrightarrow}} Cu^{+}\text{–}DTT^{*} + O_2 \underset{k_{-1}}{\overset{k_1}{\rightleftharpoons}} Cu^{+}\text{–}DTT\text{–}O_2^{*} \xrightarrow{k_2} Cu^{2+}\text{–}DTT + oxyDTT$$

$$Mn^{2+} + DTT \xrightarrow{\text{fast}} Mn^{2+}\text{–}DTT \underset{K'}{\overset{\text{electron transfer}}{\longleftrightarrow}} Mn^{+}\text{–}DTT^{*} + O_2 \underset{k_{-1'}}{\overset{k_{1'}}{\rightleftharpoons}} Mn^{+}\text{–}DTT\text{–}O_2^{*} \xrightarrow{k_{2'}} Mn^{2+}\text{–}DTT + oxyDTT$$

Equilibrium:

$$[O_2]+[Cu^{+}\text{–}DTT\text{–}O_2^{*}]+[Mn^{+}\text{–}DTT\text{–}O_2^{*}] = [O_2]_0 \quad (S8)$$

$$k_1[Cu^{+}\text{–}DTT^{*}][O_2] = k_{-1}[Cu^{+}\text{–}DTT\text{–}O_2^{*}]+k_2[Cu^{+}\text{–}DTT\text{–}O_2^{*}] \quad (S9)$$

$$k_1{'}[Mn^{+}\text{–}DTT^{*}][O_2] = k_{-1}{'}[Mn^{+}\text{–}DTT\text{–}O_2^{*}]+k_2{'}[Mn^{+}\text{–}DTT\text{–}O_2^{*}] \quad (S10)$$

$$K[Cu^{2+}\text{–}DTT] = [Cu^{+}\text{–}DTT^{*}] \quad (S11)$$

$$K{'}[Mn^{2+}\text{–}DTT] = [Mn^{+}\text{–}DTT^{*}] \quad (S12)$$

Derivation:

$$[Cu^{+}\text{–}DTT\text{–}O_2^{*}] = \frac{k_1}{k_{-1}+k_2}[O_2][Cu^{+}\text{–}DTT^{*}] = \frac{Kk_1}{k_{-1}+k_2}[O_2][Cu^{2+}\text{–}DTT] \quad (S9')$$

$$[Mn^{+}\text{–}DTT\text{–}O_2^{*}] = \frac{k_{1'}}{k_{-1'}+k_{2'}}[O_2][Mn^{+}\text{–}DTT^{*}] = \frac{K'k_{1'}}{k_{-1'}+k_{2'}}[O_2][Mn^{2+}\text{–}DTT] \quad (S10')$$

$$[O_2]\{1+\frac{Kk_1}{k_{-1}+k_2}[Cu^{2+}\text{–}DTT]+\frac{K'k_{1'}}{k_{-1'}+k_{2'}}[Mn^{2+}\text{–}DTT]\}=[O_2]_0 \quad (S13)$$

$$OP_{DTT}\ (\text{uM/min}) = \frac{-d[DTT]}{dt} = \frac{d[oxyDTT]}{dt} = k_2[Cu^{+}\text{–}DTT\text{–}O_2^{*}]+k_{2'}[Mn^{+}\text{–}DTT\text{–}O_2^{*}]$$

$$= [O_2]_0\frac{\frac{k2k1}{k\text{-}1+k2}[Cu^{2+}\text{–}DTT]+\frac{k2'k1'}{k\text{-}1+k2'}[Mn^{2+}\text{–}DTT]}{1+\frac{k1}{k\text{-}1+k2}[Cu^{2+}\text{–}DTT]+\frac{k1'}{k\text{-}1'+k2'}[Mn^{2+}\text{–}DTT]} = \frac{\frac{V_{max}}{K_{QM}}[Cu^{2+}\text{–}DTT]+\frac{V_{max}'}{K_{QM}'}[Mn^{2+}\text{–}DTT]}{1+[Cu^{2+}\text{–}DTT]/K_{QM}+[Mn^{2+}\text{–}DTT]/K_{QM'}} \quad (S14)$$

$$(k_2[O_2]_0 = V_{max}, K_{QM} = \frac{k_{-1}+k_2}{Kk_1};\ k_{2'}[O_2]_0 = V_{max'}, K_{QM'} = \frac{k_{-1'}+k_{2'}}{K'k_{1'}})$$

Approximation:

$$[Cu^{2+}\text{–}DTT]=[Cu^{2+}]_0\ ([DTT]_0 \gg [Cu]_0) \quad (S15)$$

$$[Mn^{2+}\text{–}DTT]=[Mn^{2+}]_0\ ([DTT]_0 \gg [Mn]_0) \quad (S16)$$

Result:

$$OP_{Cu\&Mn}\ (\text{uM/min}) = \frac{\frac{V_{max}}{K_{QM}}[Cu^{2+}]_0+\frac{V_{max}'}{K_{QM}'}[Mn^{2+}]_0}{1+[Cu^{2+}]_0/K_{QM}+[Mn^{2+}]_0/K_{QM'}} \quad (S17)$$

Index of mixture effect:

$$I_{ME,\,Mn\&Cu}=\frac{\dfrac{\dfrac{V_{max}}{K_{QM}}[Cu^{2+}]_0+\dfrac{V_{max}'}{K_{QM}'}[Mn^{2+}]_0}{1+[Cu^{2+}]_0/K_{QM}+[Mn^{2+}]_0/K_{QM'}}}{\dfrac{V_{max}[Cu^{2+}]_0}{K_{QM}+[Cu^{2+}]_0}+\dfrac{V_{max'}[Mn^{2+}]_0}{K_{QM'}+[Mn^{2+}]_0}} \qquad (S18)$$

Table S1. Statistics of $PM_{2.5}$ and chemical species in ambient samples

	$PM_{2.5}$ (μg/m^3)	WS-Mn (ng/m^3)	WS-Fe (ng/m^3)	WS-Cu (ng/m^3)	WSOC (μg C/m^3)
Mean	55.60	17.21	24.03	4.82	5.47
STDEV	42.17	10.01	16.77	3.02	2.63
MIN	8.12	0.99	2.66	0.70	2.11
25%	25.91	10.77	11.92	2.58	3.53
50%	43.30	16.45	20.06	4.01	5.16
75%	73.47	23.61	32.44	7.32	6.23
MAX	192.31	44.07	76.76	11.99	15.75

Table S2. Chemical data of four selected $PM_{2.5}$ samples (μg/m^3)

	$PM_{2.5}$	Na^+	Cl^-	NH_4^+	K^+	Ca^{2+}	Mg^{2+}	MSA^-	NO_3^-	$SO_4^=$	$C_2O_4^=$
BJ160719	67.7	0.156	0.843	11.330	0.507	0.225	0.000	0.033	18.415	17.288	0.452
BJ160923	95.2	0.242	0.623	14.710	0.820	0.143	0.000	0.103	30.527	16.135	0.571
BJ161104	170.7	0.425	3.522	16.348	1.599	0.163	0.006	0.191	28.511	19.798	0.432
BJ161204	136.6	0.520	4.388	12.397	1.383	0.838	0.087	0.102	23.186	12.351	0.310

	Cu	Mn	Fe	Al	Si	K	Ca	Ti	V	Zn	Pb
BJ160719	0.013	0.084	0.875	0.220	0.404	0.491	0.219	0.023	0.002	1.371	0.046
BJ160923	0.028	0.042	0.577	0.285	0.452	0.746	0.280	0.024	0.000	0.209	0.070
BJ161104	0.041	0.081	1.495	0.752	1.461	2.108	0.862	0.070	0.003	0.456	0.180
BJ161204	0.031	0.083	1.499	1.075	2.478	1.842	1.479	0.084	0.000	0.299	0.140

	WS-Cu	WS-Mn	WS-Fe	WSOC	HULIS	OC_TOR	EC_TOR	OC_TOT	EC_TOT
BJ160719	0.005	0.025	0.054	3.0	0.772	4.853	2.430	5.848	1.255
BJ160923	0.010	0.019	0.077	6.0	2.938	8.416	4.402	8.825	4.376
BJ161104	0.008	0.030	0.054	11.2	8.063	28.280	10.250	34.023	5.534
BJ161204	0.008	0.028	0.047	12.6	9.363	32.202	8.748	38.401	6.081

Table S3. Chemical composition and OP in simulated mix metal system (n=34)

Cu (μM)	Mn (μM)	Cu/Mn	Predicted OP	Observed OP	Cu (μM)	Mn (μM)	Cu/Mn	Predicted OP	Observed OP
0.112	0.013	8.825	0.427	0.477	0.101	0.233	0.433	0.485	0.440
0.056	0.006	8.825	0.339	0.452	0.020	0.058	0.346	0.273	0.249
0.028	0.003	8.825	0.240	0.294	0.050	0.233	0.216	0.452	0.351
0.014	0.002	8.825	0.152	0.212	0.020	0.116	0.173	0.333	0.297
0.007	0.001	8.825	0.087	0.157	0.002	0.016	0.145	0.076	0.117
0.202	0.058	3.461	0.489	0.468	0.005	0.032	0.145	0.137	0.186
0.101	0.058	1.730	0.432	0.397	0.009	0.064	0.145	0.227	0.264
0.202	0.116	1.730	0.498	0.448	0.023	0.160	0.145	0.375	0.418
0.050	0.058	0.865	0.362	0.324	0.046	0.319	0.145	0.479	0.538
0.101	0.116	0.865	0.452	0.397	0.020	0.233	0.087	0.416	0.275
0.202	0.233	0.865	0.516	0.519	0.036	0.517	0.069	0.527	0.486
0.118	0.140	0.840	0.470	0.524	0.018	0.259	0.069	0.427	0.394
0.059	0.070	0.840	0.386	0.453	0.009	0.129	0.069	0.309	0.350
0.029	0.035	0.840	0.284	0.335	0.004	0.065	0.069	0.199	0.130
0.015	0.018	0.840	0.186	0.254	0.002	0.032	0.069	0.116	0.080
0.007	0.009	0.840	0.110	0.151	0.001	0.016	0.069	0.063	0.049
0.050	0.116	0.433	0.398	0.313	0.001	0.008	0.069	0.033	0.001

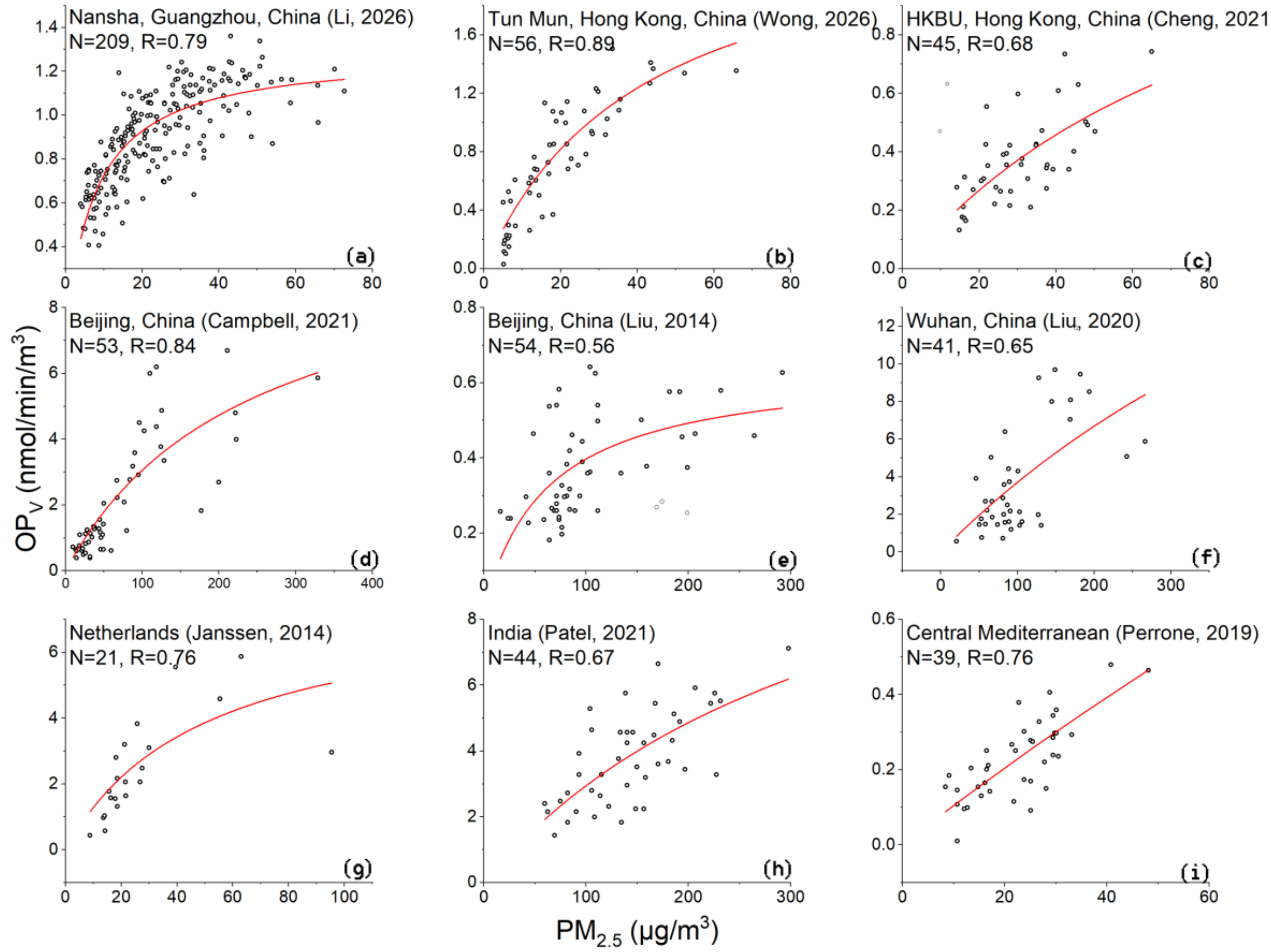

Figure S1. Relationship between OP_{DTTv} and $PM_{2.5}$ concentration in ambient samples from varied studies[1-7] (a,b are two papers from our group currently under preparation)

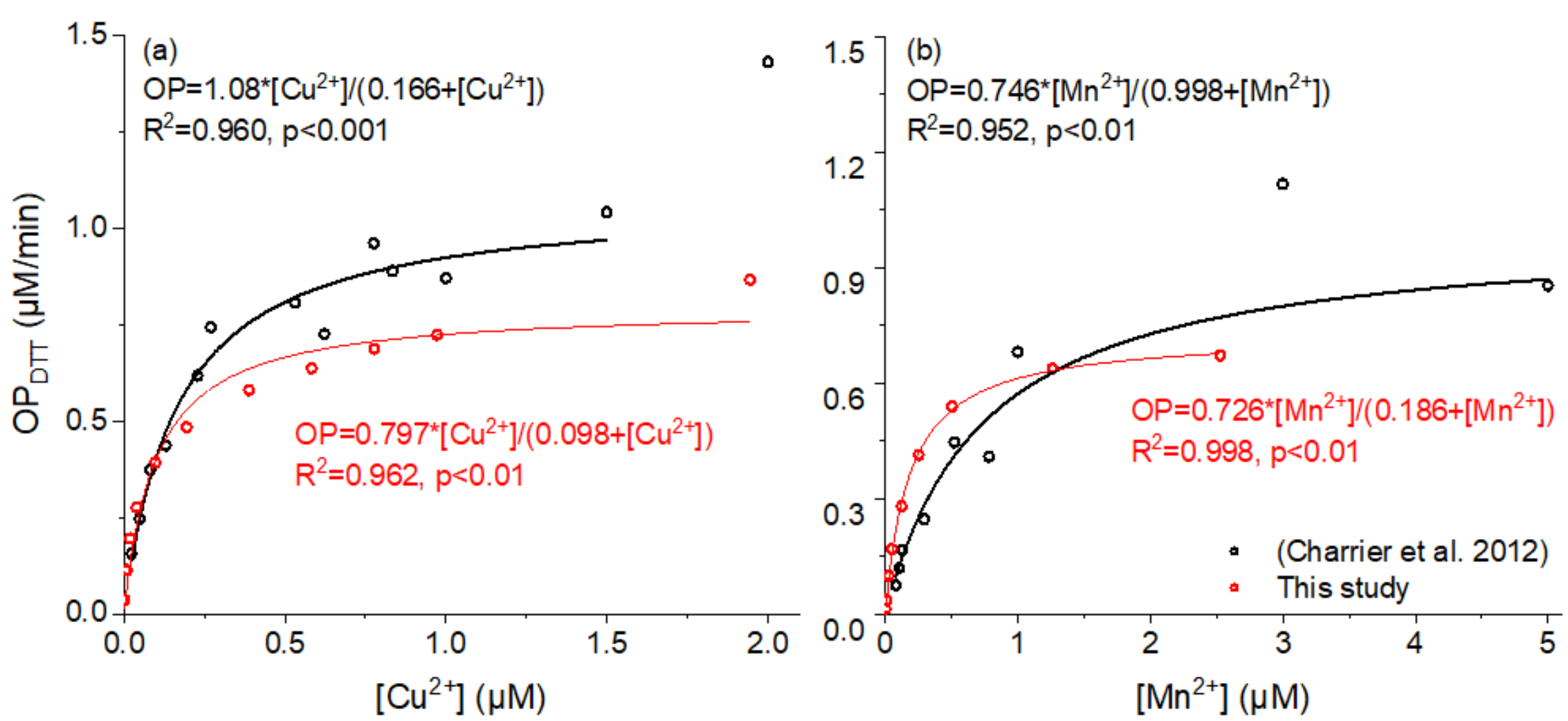

Figure S2. Metal concentration-OP_{DTT} response relationship curve (black dot is from Charrier et al.[8] and red dot is from this study)

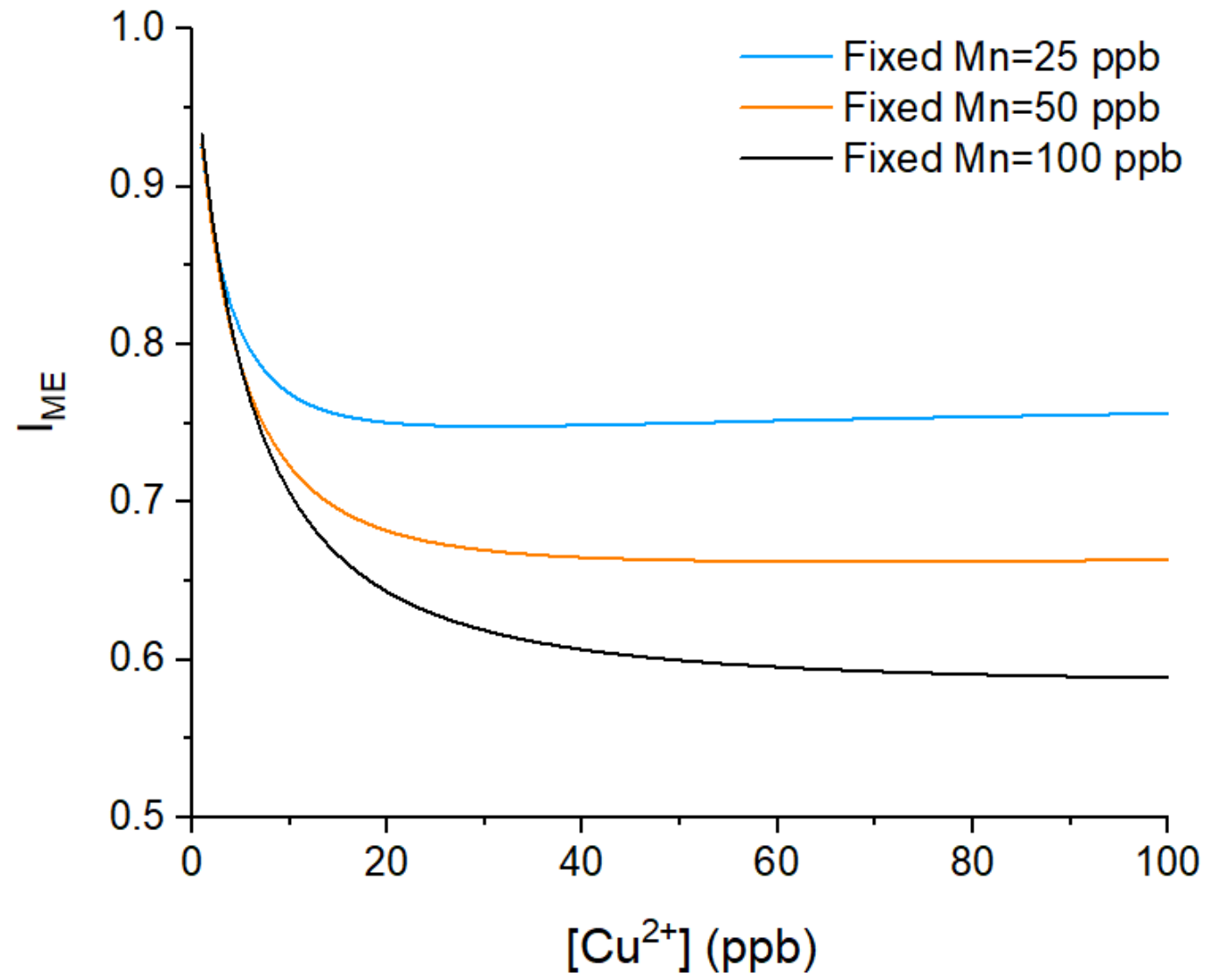

Figure S3. Calculated I_{ME} varies with different Cu concentrations at three fixed Mn concentrations (line in different colors)

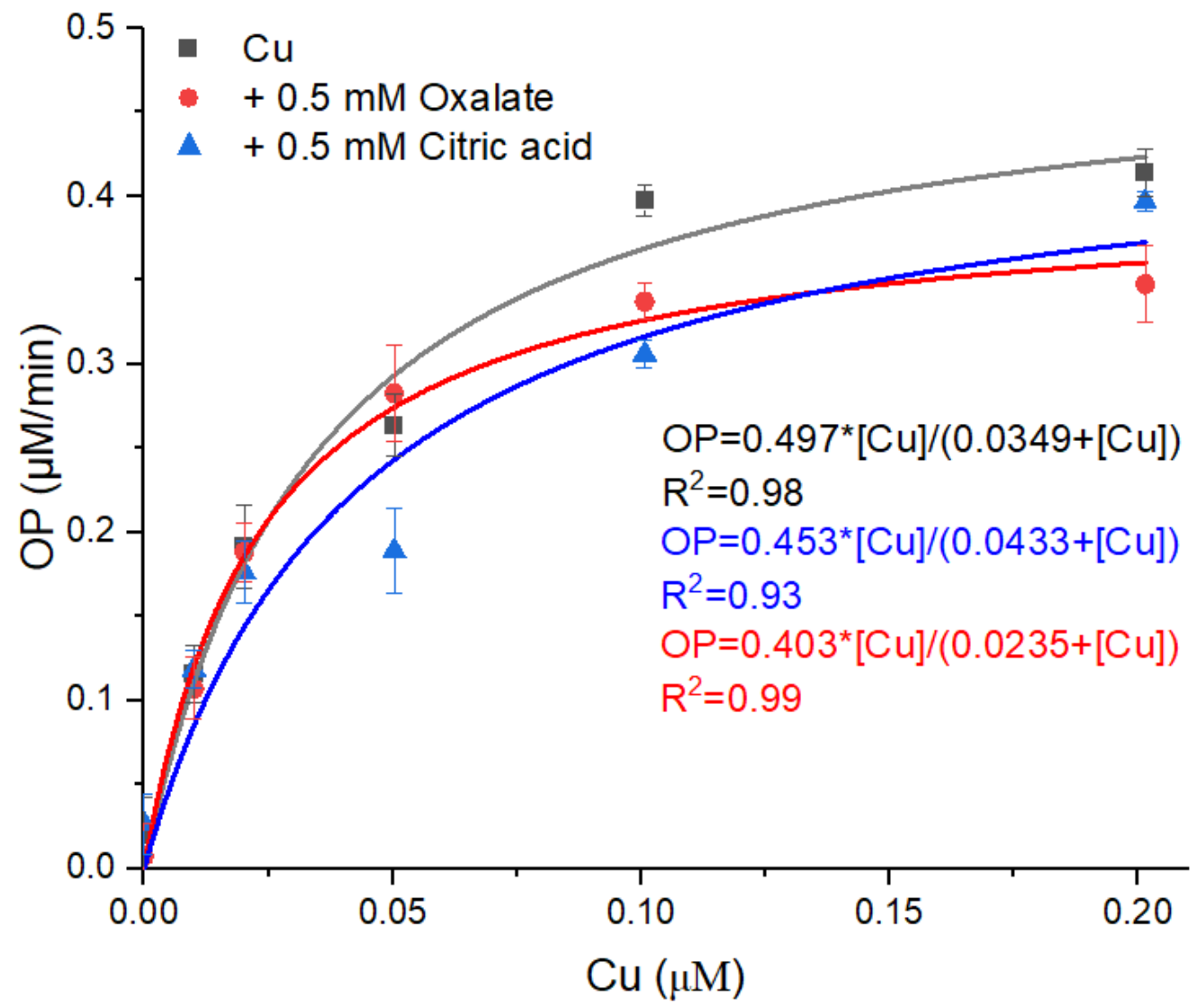

Figure S4. Relation between Cu concentration-OP_{DTT} response with/without carboxyl ligand organics (oxalate and citric acid)

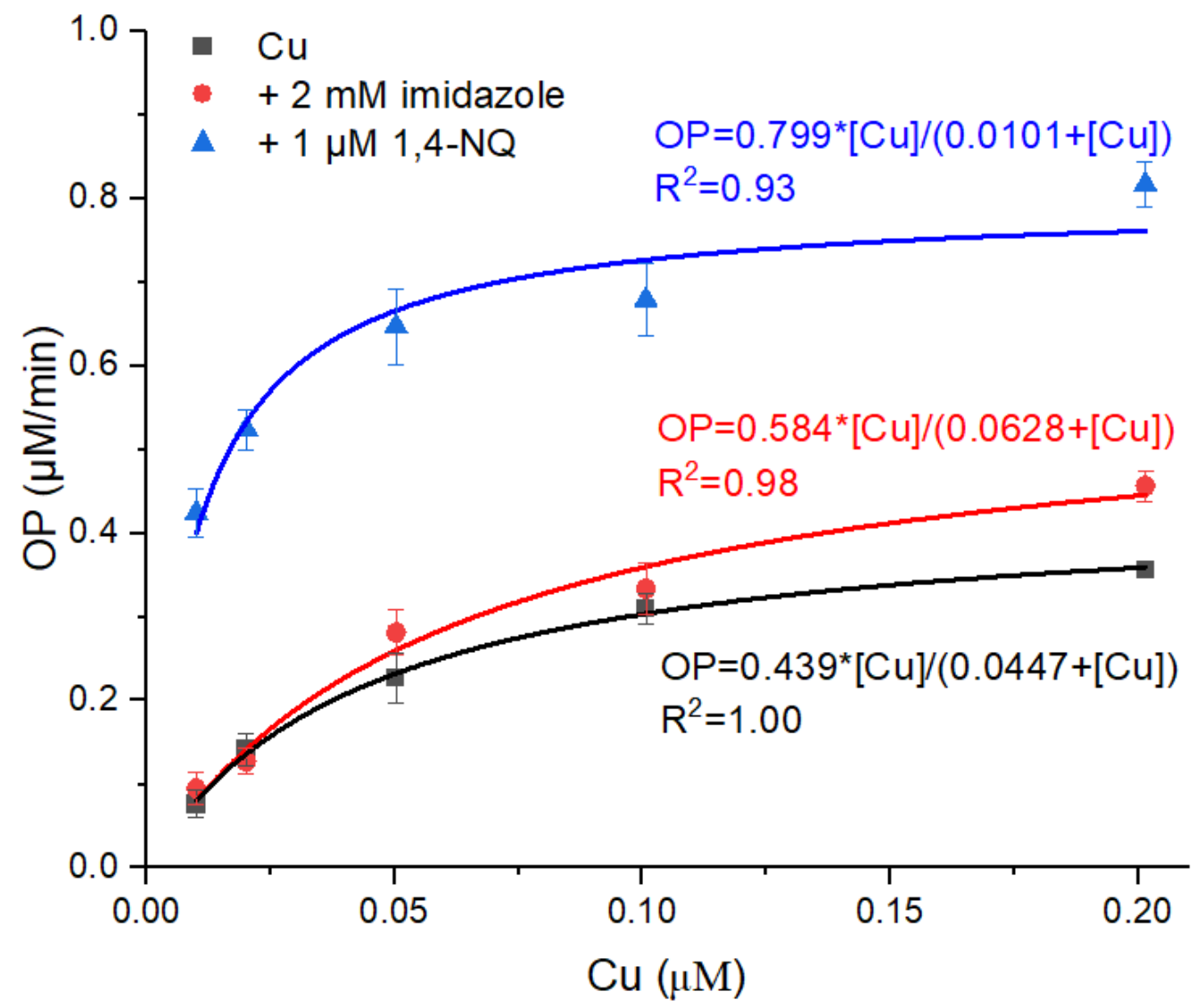

Figure S5. Relation between Cu concentration-OP_{DTT} response with/without a Nitrogen-containing organic ligand (imidazole) and a redox-active organic compound (1,4-Naphthoquinone).

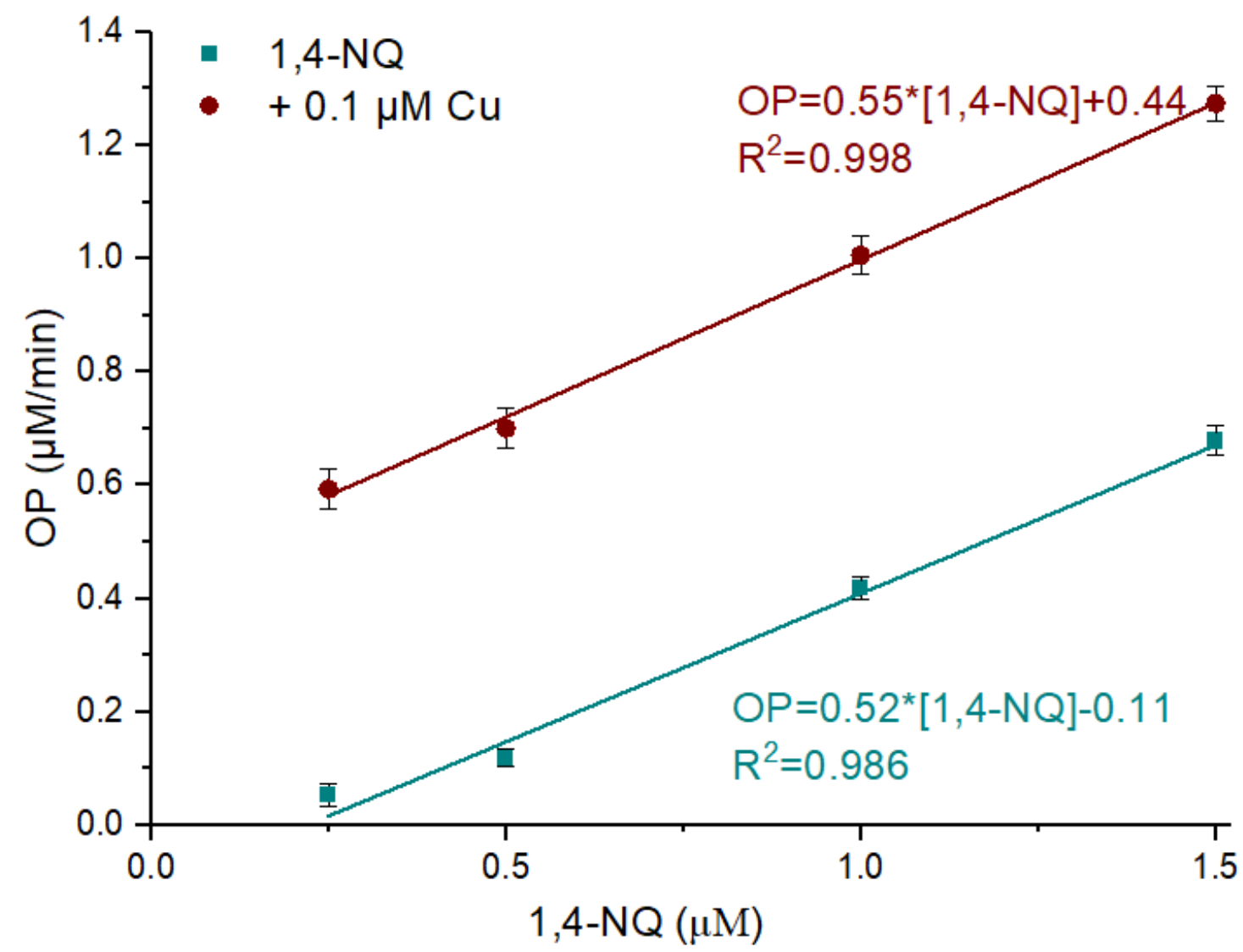

Figure S6. Linear relation between 1,4-NQ concentration-OP_{DTT} response with/without Cu

References:

1. Cheng, Y.; Ma, Y.; Dong, B.; Qiu, X.; Hu, D., Pollutants from primary sources dominate the oxidative potential of water-soluble PM2.5 in Hong Kong in terms of dithiothreitol (DTT) consumption and hydroxyl radical production. *J. Hazard. Mater.* **2021,** *405*, 124218.
2. Perrone, M. R.; Bertoli, I.; Romano, S.; Russo, M.; Rispoli, G.; Pietrogrande, M. C., PM2.5 and PM10 oxidative potential at a Central Mediterranean Site: Contrasts between dithiothreitol- and ascorbic acid-measured values in relation with particle size and chemical composition. *Atmospheric Environment* **2019,** *210*, 143-155.
3. Patel, A.; Satish, R.; Rastogi, N., Remarkably High Oxidative Potential of Atmospheric PM2.5 Coming from a Large-Scale Paddy-Residue Burning over the Northwestern Indo-Gangetic Plain. *ACS Earth and Space Chemistry* **2021**.
4. Liu, Q. Y.; Lu, Z. J.; Xiong, Y.; Huang, F.; Zhou, J. B.; Schauer, J. J., Oxidative potential of ambient PM2.5 in Wuhan and its comparisons with eight areas of China. *Science of the Total Environment* **2020,** *701*.
5. Campbell, S. J.; Wolfer, K.; Utinger, B.; Westwood, J.; Zhang, Z. H.; Bukowiecki, N.; Steimer, S. S.; Vu, T. V.; Xu, J.; Straw, N.; Thomson, S.; Elzein, A.; Sun, Y.; Liu, D.; Li, L.; Fu, P.; Lewis, A. C.; Harrison, R. M.; Bloss, W. J.; Loh, M.; Miller, M. R.; Shi, Z.; Kalberer, M., Atmospheric conditions and composition that influence PM2.5 oxidative potential in Beijing, China. *Atmospheric Chemistry and Physics* **2021,** *21*, (7), 5549-5573.
6. Liu, Q. Y.; Baumgartner, J.; Zhang, Y. X.; Liu, Y. J.; Sun, Y. J.; Zhang, M. G., Oxidative Potential and Inflammatory Impacts of Source Apportioned Ambient Air Pollution in Beijing. *Environmental Science & Technology* **2014,** *48*, (21), 12920-12929.
7. Janssen, N. A. H.; Yang, A. L.; Strak, M.; Steenhof, M.; Hellack, B.; Gerlofs-Nijland, M. E.; Kuhlbusch, T.; Kelly, F.; Harrison, R. M.; Brunekreef, B.; Hoek, G.; Cassee, F., Oxidative potential of particulate matter collected at sites with different source characteristics. *Science of the Total Environment* **2014,** *472*, 572-581.
8. Charrier, J. G.; Anastasio, C., On dithiothreitol (DTT) as a measure of oxidative potential for ambient particles: evidence for the importance of soluble transition metals. *Atmospheric Chemistry and Physics* **2012,** *12*, (19), 9321-9333.
